# Peripheral sensory neurons govern development of the nervous system in bivalve larvae

**DOI:** 10.1186/s13227-019-0133-6

**Published:** 2019-09-12

**Authors:** Olga V. Yurchenko, Anna V. Savelieva, Natalia K. Kolotuchina, Elena E. Voronezhskaya, Vyacheslav A. Dyachuk

**Affiliations:** 1 National Scientific Center of Marine Biology, Far Eastern Branch, Russian Academy of Sciences, Vladivostok, 690041 Russia; 2 N.K. Koltsov Institute of Developmental Biology, Russian Academy of Sciences, Moscow, 119991 Russia; 3 Department of Neuroscience, Karolinska Institutet, Stockholm, Sweden; 4 Department of Nanophotonics and Metamaterials, ITMO University, St. Petersburg, Russia

**Keywords:** Mollusk, Neurotransmitters, Nerve cords, Ganglia, Tetraneuralia

## Abstract

Recent findings regarding early lophotrochozoan development have altered the conventional model of neurogenesis and revealed that peripheral sensory elements play a key role in the initial organization of the larval nervous system. Here, we describe the main neurogenetic events in bivalve mollusks in comparison with other Lophotrochozoa, emphasizing a novel role for early neurons in establishing larval nervous systems and speculating about the morphogenetic function of the apical organ. We demonstrate that during bivalve development, peripheral sensory neurons utilizing various transmitters differentiate before the apical organ emerges. The first neurons and their neurites serve as a scaffold for the development of the nervous system. During veliger stage, cerebral, pleural, and visceral ganglia form along the lateral (visceral) nerve cords in anterior-to-posterior axis. The pedal ganglia and corresponding ventral (pedal) nerve cords develop much later, after larval settlement and metamorphosis. Pharmacological abolishment of the serotonin gradient within the larval body disrupts the navigation of “pioneer” axons resulting in malformation of the whole nervous system architecture. Comparative morphological data on neurogenetic events in bivalve mollusks shed new light on the origin of the nervous system, mechanisms of early axon navigation, and sequence of the tetraneurous nervous system formation. Furthermore, this information improves our understanding of the basic nervous system architecture in larval Bivalvia and Mollusca.

## Unsolved questions on lophotrochozoans neurodevelopment

Lophotrochozoa, a morphologically varied group of animals, include worm-like forms, shelled animals, and soft-bodied slugs, which makes searching for common anatomical and morphological features in adults daunting and unsuccessful to date. However, during lophotrochozoan early larval development, ancestral morphological and molecular patterns are present with formation of the nervous system being among the most well-conserved and prominent features of this development. Therefore, development of the nervous system has long attracted the attention of comparative biologists for testing evolutionary and phylogenetic hypotheses.

Although for more than 100 years, researchers have attempted to understand the details and general patterns of this development utilizing available methodology [1–6], some major questions remain concerning the following points: (1) homologous characters that have been reduced or strongly modified in adults by adaptation (for example, simplification of the organization of the nervous system); (2) phylogenetic relationships among lophotrochozoans and their kinship with other groups of animals (Ecdysozoa and Deuterostomia); and (3) the potential body plan (bauplan) of the last common ancestor for particular groups. Answering these questions requires a variety of methodological approaches, including morphological/physiological and molecular methods.

Bivalves are seldom used in studies of lophotrochozoan neurodevelopment. These animals, with sedentary or free-living lifestyles as adults, develop from free-swimming larvae that hatch as trochophores, develop through the veliger stage, undergo metamorphosis, and then settle [1–4, 6].

### Adult and larval neuroanatomy: correction of morphological misinterpretations

**Fig. 1:**
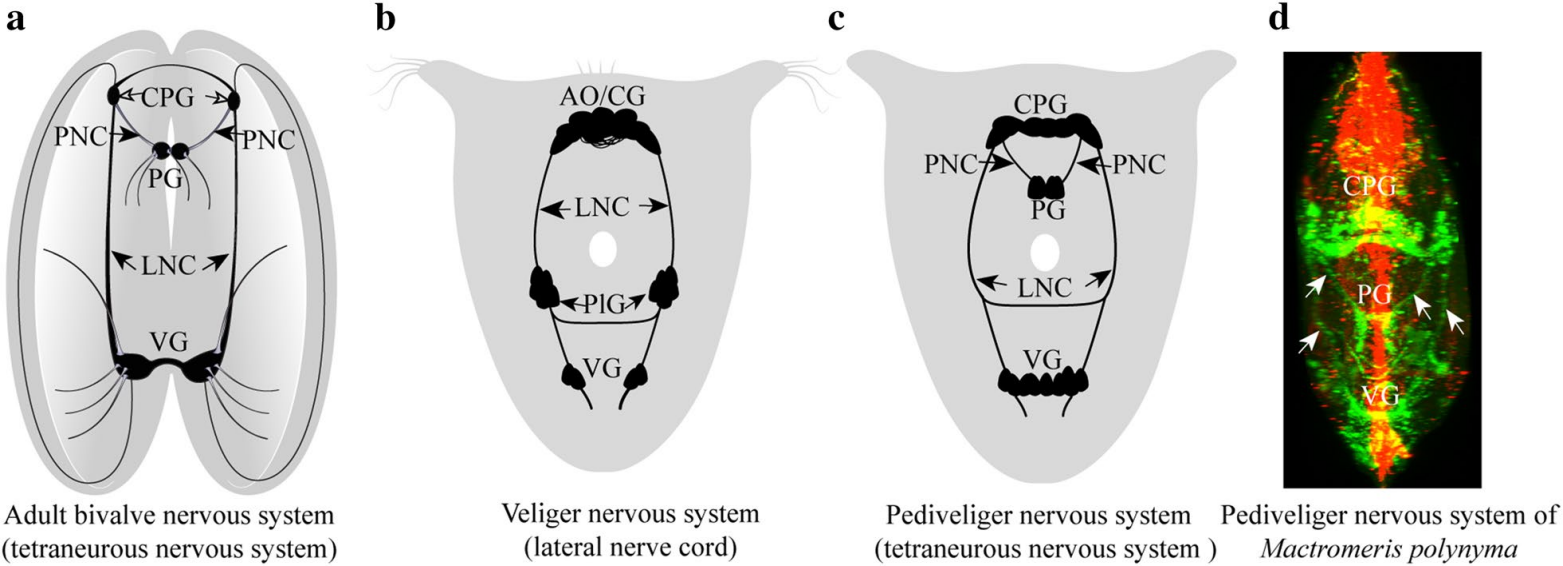
Architecture of the nervous system of adult bivalves (**a**) and larvae before and after metamorphosis (**b**–**d**). **a** Tetraneurous nervous system (ventral + pedal cords) of the adult bivalve. **b** Nervous system of bivalve larvae. **c** The tetraneurous bivalve nervous system after settlement/metamorphosis. **d** Nervous system of a mactrid juvenile *Mactromeris polynyma* from plankton as visualized by whole-mount immunostaining for FMRFamide (green) and acetylated alpha-tubulin (red) (the arrows indicate the lateral nerve cord and ventral (pedal) nervous cords). *CPG* cerebropleural ganglion, *PNC* pedal nervous cord, *PG* pedal ganglion, *LNC* lateral nerve cord, *VG* visceral ganglion, *AO/CG* apical organ/cerebral ganglion, *PlG* pleural ganglion

Previous investigations on bivalves have concluded that the central nervous system (CNS) of adults consists of paired cerebropleural and visceral ganglia, the latter typically fused and connected to the cerebropleural ganglia by longitudinal lateral cords [6–12] (Fig. 1a). These neurostructures innervate most internal organs. Fused pedal ganglia belong to CNS and innervate the foot, and are connected to the cerebropleural ganglia via the ventral (pedal) cords (Fig. 1a) [4, 13]. Accordingly, the nervous system of adult bivalves is tetraneural, consisting of two prominent pairs of longitudinal neurite bundles (cords) ganglionated to various extents [4, 6–13].

Research on the developmental neurogenesis of bivalves is limited and interpretation of larval morphology often contradictory [3, 4, 6, 14, 15]. Findings published to date do not allow for an exact phylogenetic placement of bivalves within Mollusca or the evolutionary origin of the last common ancestor of bivalves and the other molluskan taxa to be determined. A number of evolutionary hypotheses concerning the origin of bivalves and their relationship to potential sister groups within the mollusks (Testaria, Conchifera, Diasoma), as well as the relationship of mollusks to other lophotrochozoan taxa (the annelid-mollusk and mollusk-entoproct hypotheses) have been proposed [4, 16–20]. However, available developmental data provide no clear support for any of these hypotheses. Moreover, the neurogenetic mechanisms by which the ganglia develop along the nerve cords or organize into a two-ring tetraneural structure remain unclear.

In particular, the variability of early neurogenetic events in bivalve species [3, 14, 15, 21] and the lack of information on the late stages of development are the major barriers at present to fully understand neurogenesis in mollusk larvae. Nevertheless, studies have shown that bivalve larvae have a ganglionic morphology of the nervous system and paired ganglia (cerebral and visceral) lie along the lateral (visceral) nerve cord in veliger larvae of mytilids [3, 22], ostreids [14, 22–24], and mactrids [25] Additional paired ganglia, identified as pedal ganglia sitting on lateral nerve cords in these same larvae, are very probably the same as the pleural ganglia detected earlier in larvae of *Ostrea edulis*, *Crassostrea virginica*, and *Mytilus galloprovincialis* [1, 23, 26].

Accordingly, although morphology varies, the nervous system of bivalve veligers consists of three paired ganglia (cerebral, pleural, and visceral) connected by the lateral cord (Fig. 1b). However, larval neuromorphology also differs from the adult nervous system with respect to the degree of fusion of the cerebral and pleural ganglia, indicating that this ganglionic fusion occurs during late development. As published previously [13, 27], cerebral and pleural ganglia develop independently in some mollusk larvae and only fuse in the adults (e.g., in *Dreissena*, *Mactra*, and *Arca pernoides*. Location and timing of the development of paired pedal ganglia remain unknown. Researchers schematically depict pedal ganglia and their connections with cerebral ganglia, emphasizing that in larvae, paired pedal (ventral) and lateral (visceral) nerve cords together build a tetra-cord structure [4, 6, 20]. Nonetheless, no detailed description of the development of the pedal nervous system has yet appeared.

Here, we first review the findings on later developmental stages (after the settlement and metamorphosis of pediveligers), when pedal ganglia with their cerebropleuro-pedal connections (pedal cords) arise (Fig. 1c). Immunostaining of larvae (caught in plankton) during the late stages of development revealed that the pedal ganglia develop during late ontogeny only (Fig. 1d) and subsequently fuse later as shown for other bivalves [13]. Altogether, the early set of paired ganglia, including cerebral, pleural, and visceral, is more fully developed in the veliger stages, while the ventral part of the nervous system (paired pedal ganglia) differentiate later on (Fig. 1c, d), in association with the foot formation and functions. Thereafter, we consider the underlying cellular mechanisms in more detail, including how the various parts of the nervous system are formed during early development of bivalve mollusks.

### Early events in bivalve neurodevelopment

It is generally accepted that the first neurons to appear in larvae are sensory cells, responsible for converting external stimuli from the environment and appropriately regulating larval behavior and development in Lophotrochozoa [28–30], Echinodermata [31, 32], and Cephalochordata [33]. Most commonly, the apical organ (AO) is the first sensory structure to appear. This organ consists of neurons that employ several different transmitters to regulate the rate of development and locomotion of larvae as well as settlement and metamorphosis [30, 34–36].

However, some evidence indicates that simultaneously with or even before the neurotransmitter expression in AO cells, solitary sensory cells appear on the periphery of the body of several trochozoan larvae. The function of these pioneer sensory neurons remains unknown. One hypothesis is that they help to provide scaffolding for the definitive nervous system [14, 21, 37–39].

Recent studies on the bivalve mollusk *Crassostrea gigas* confirm the early appearance of peripheral sensory neurons [14] and, together with the experimental findings on *Mytilus trossulus,* larvae presented here indicate the morphogenetic significance of these cells in connection with formation of the nervous system.

**Fig. 2:**
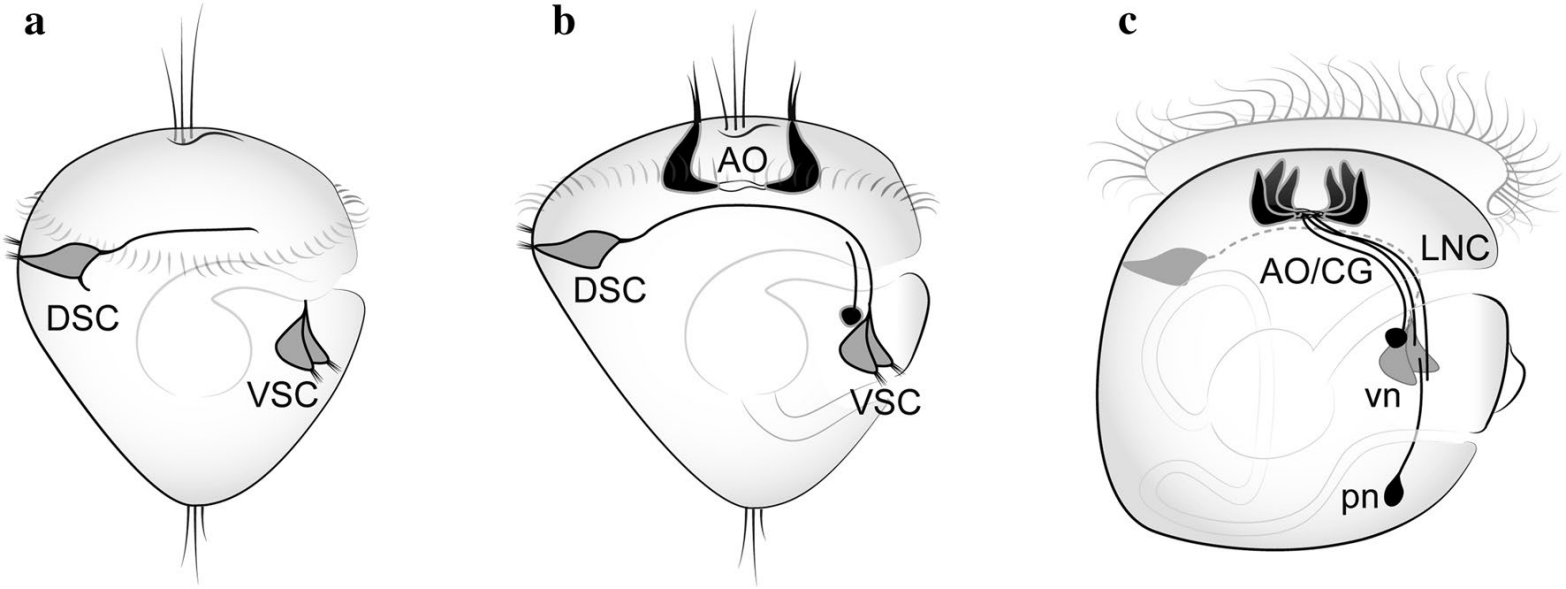
Neurogenesis of the oyster *Crassostrea gigas*: FMRF-immunoreactive nervous system. **a** Early neurons that appear in the dorsal (DSC) and ventral (VSC) peripheral sensory centers, before appearance of transmitters in the apical organ (AO). **b**, **c** They provide morphogenetic scaffolding for the anlagen of the lateral nerve cords (LNC) during oyster development. *vn* ventral neurons, *pn* posterior neurons (Schematic drawings adapted by Olga Kharchenko from Yurchenko et al. [14])

In greater detail, two groups of sensory cells can be detected on the dorsal and ventral sides of the trochophore larvae of the oyster *C. gigas* prior to the emergence of the AO. The neurites of the FMRFamide-immunoreactive (FMRF-ir) neurons in the dorsal sensory group (dorsal sensory center, DSC) extend towards the neurons of the ventral sensory group (ventral sensory center, VSC) (Fig. 2a). As neurogenesis proceeds, these long axons of the dorsal neurons terminate near the ventral cells and these interconnections are organized into paired dorso-ventral cords (Fig. 2b). However, there is no direct connection to the differentiating cells of the AO located between the dorso-ventral cords [14].

At later stages, the AO and neurons located posteriorly extend their processes to the ventral cells and along the dorso-ventral cord. Thus, in the veliger of *C. gigas*, the anlagen of the lateral nerve cords have already been formed (Fig. 2c). Interestingly, the early dorsal and ventral sensory centers are transient, and their cells and processes cease to express immunoreactivity and probably disappear during the veliger stage. However, the questions whether the cells of the ventral sensory centers are precursors of the pleural ganglia and the posterior cells are precursors of the visceral ganglia remain unanswered.

We speculate that early peripheral sensory cells provide scaffolding for the differentiating nervous system, marking the location for subsequent cell migration and organization of the major central ganglia and nerve cords. The exact sources of neuronal progenitors during lophotrochozoan development are largely unknown. Some data indicate that cells in cerebral placodes give rise to neurons within the cerebral ganglia [40, 41]. Several mechanisms for the differentiation of ganglionic neurons have been proposed, involving local nerve-dependent signals [42], migration of neuronal precursors from outside into ganglia anlagen [43], and/or the glial or sensory epithelium (unpublished data). However, none of these hypotheses has experimental support to date.

### The transmitters employed by the primary sensory neurons

At present, we have no explanation why serotonin (5-HT) and FMRFamide-related peptides (FMRF-RP) are the primary transmitters expressed by the first sensory neurons that appear during bivalve development. FMRFamide and related peptides, in addition to 5-HT, influence multiple physiological processes, including heart rate, blood pressure, motility of the gut, feeding, and locomotory behavior in both adult and larval invertebrates [44–47]. The peripheral sensory neurons in the larvae of different lophotrochozoan groups contain FMRF-RP (gastropod mollusks [48–50]) or 5-HT and FMRF-RP in different neurons (annelids [51] and bivalve mollusks [3, 14]) or in one and the same neuron (polyplacophoran mollusk [52] and annelids [53]).

Neurons with different immunoreactivity (FMRF-RP and 5-HT) innervating several larval organs may perform common functions, e.g., affecting the beating of cilia or innervating muscles [22, 54–58]. Thus, both may be involved in the regulation of locomotion, distribution, and defensive behavior. In addition, the FMRFamide peptide family modulates ligand-gated channels that transport Na^+^ and associated water, rendering larvae resistant to osmotic pressure [29, 59–62]. FMRFamide, expressed in the early peripheral neurons of bivalve and gastropod larvae [3, 14, 21, 48], has been suggested to modulate their behavior in response to environmental changes (for example, in salinity, temperature, and pH) in their ecological niche, where appropriate movement in the water column determines successful survival. However, more experiments on the role of early sensory neurons in larval development are clearly required.

### Neurites of sensory neurons play important roles in morphogenetic events

**Fig. 3:**
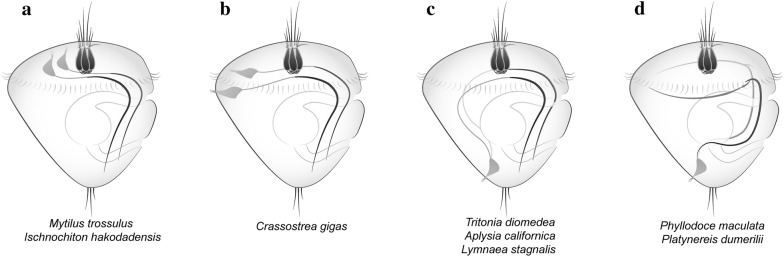
Schematic depiction of the location of early sensory elements in representative members of different lophotrochozoan groups. The long axons of early peripheral neurons (gray color) are arranged similarly in two parallel nerve bundles along the ventral side of the larvae, where the anlagen of the lateral nerve cords will later form. **a** Larvae of the mussel *Mytilus trossulus* and the chiton *Ischnochiton hakodadensis*; **b** Larva of the oyster *Crassostrea gigas*; **c** Larva of the gastropods *Tritonia diomedea*, *Aplysia californica*, and *Lymnaea stagnalis*; **d** Larva of the annelids, *Phyllodoce maculata* and *Platynereis dumerilii*

Neurogenesis in several groups of Lophotrochozoa has been found to begin with the emergence of peripheral neurons. Importantly, the locations of these early larval neurons vary in different species. In the bivalve mollusk *Mytilus trossulus*, they appear in the episphere and their processes organize into two nerve bundles that run in parallel along the ventral side of the larvae [3] (Fig. 3a). In the polyplacophora *Ischnochiton hakodadensis*, the bodies of these early cells are located in the episphere and their long axons extend underneath the neuropil of the AO and thereafter caudally in two bundles on the ventral side [48] (Fig. 3a).

Recently, we demonstrated that in the trochophore larvae of *C. gigas,* early FMRF-ir peripheral cells differentiate in the hyposphere and send their long axons from the dorsal to the ventral side [14] (Fig. 3b). In the gastropod mollusks *Tritonia diomedea*, *Aplysia californica*, and *Lymnaea stagnalis*, peripheral cells are located caudally in the hyposphere and their processes run anteriorly along the curvature of the larval body towards the AO and then turn ventrally [21, 37, 50, 63, 64] (Fig. 3c). In the polychaetes *Phyllodoce maculata* and *Platynereis dumerilii*, the solitary early cells are located in the most caudal region of the hyposphere, their long axon bifurcating close to the cell body with two long processes turning to the ventral side and running anteriorly [51, 53] (Fig. 3d).

All early peripheral cells bear cilia and are considered to be sensory [21]. The prominent features of the early sensory peripheral cells in the larval body are their long axons. Independent of the location of the perikaryon, episphere or hyposphere, dorsally or ventrally, the emerging axons extend towards and follow the ventral side in two parallel longitudinal lateral paired cords. Thus, the pioneer processes ultimately delicate a position or configuration for future developed lateral nerve cords, which form by several pathways (dorso-ventral way or anterior–posterior way, or posterior–anterior way). In general, longitudinal nerve cords are a common feature for non-lophotrochozoan and lophotrochozoan protostomes, evolutionarily ancient and can be considered to be an ancestral character of Bilateria.

### The role of serotonin in the navigation of the axons of early neurons

During development, the navigation and guidance of dendritic and axonal extensions and connections between neurons and non-neuronal targets are strictly regulated by a multitude of growth and transcriptional factors, adhesion molecules, and target receptors [65, 66]. Among the well-studied factors, 5-HT has been found to be a navigational cue during neural development in vertebrates and invertebrates [67, 68].

In the gastropod mollusk *Helisoma*, 5-HT and dopamine inhibit the elongation of neurites of specific identified neurons [69–71], while the initial phase of the process, 5-HT can also inhibit neurite growth [71, 72].

During early larval development, the primary source of 5-HT is the apical organ (AO), where specific neurons secrete transmitter via their varicose neuropil [73]. Apical restriction of secretion in this manner results in a gradient of 5-HT along the anterior–posterior axis of the larval body and this may help to navigate the axons of peripheral sensory neurons. Clearly, application of 5-hydroxytryptophan (5-HTP), the precursor of 5-HT, to trochophores of the annelid *Platynereis dumerilii* increases levels of 5-HT within AO neurons specifically and influences the rate of larval development without altering the structure of the major nerve elements. In contrast, application of 5-HT itself, increasing the concentration of this transmitter throughout the larval body and thereby abolishing the gradient, causes irreversible structural damage to the larval nervous system and, ultimately, larval death [21].

**Fig. 4:**
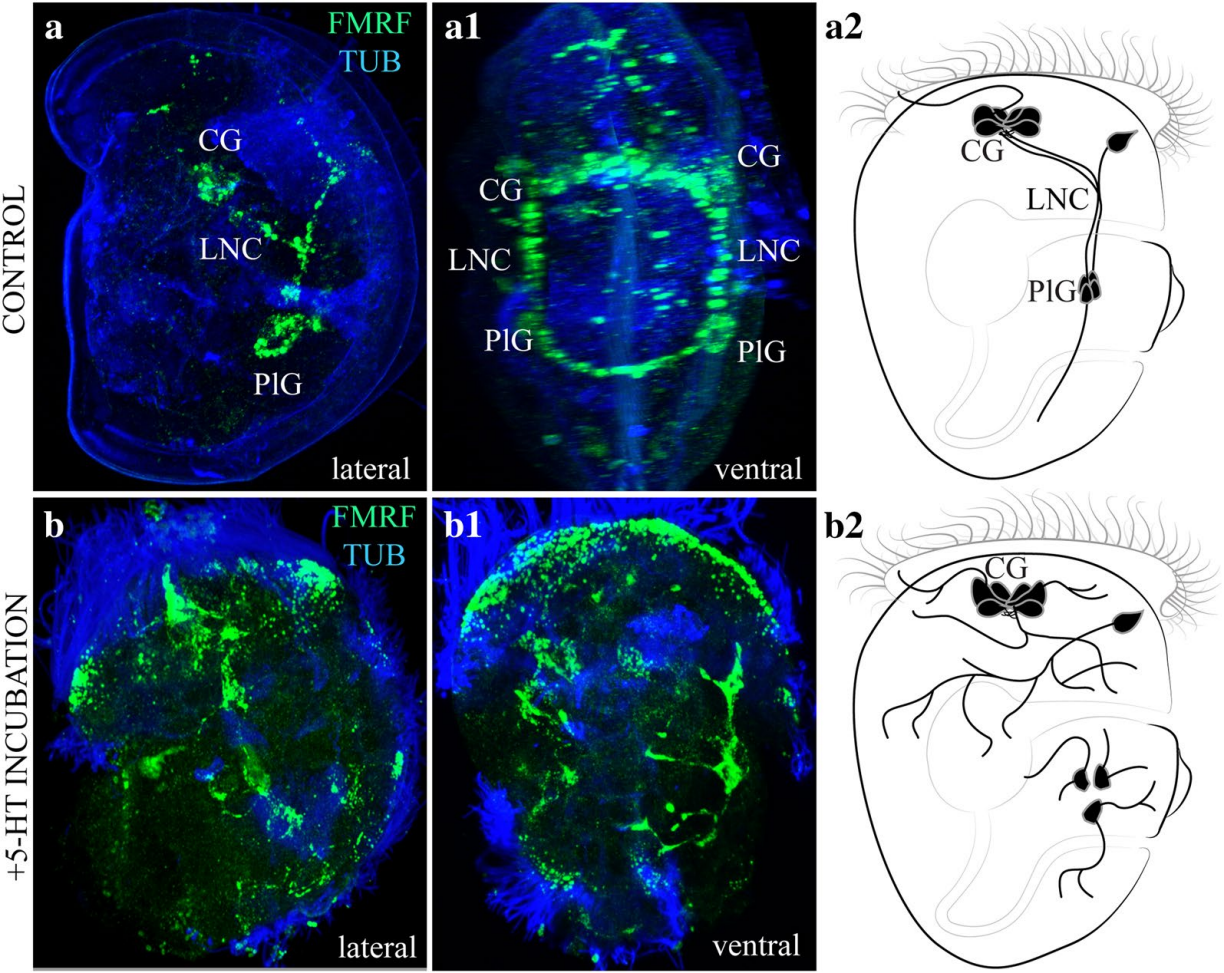
Effect of serotonin on the growth of FMRFamide-immunoreactive processes during development of the mollusk *Mytilus trossulus*. Mussel trochophores (32 hpf) were cultured in filtered seawater in the presence of 5-HT (10^−6^ M) or without any additives (the control). Larvae were collected at the veliger stage (60 hpf) and subjected to whole-mount immunostaining for FMRFamide (green) and acetylated alpha-tubulin (blue) as described earlier [14]. **a**–**b2** right lateral view of untreated (**a**, **a2**) and 5-HT-treated (**b**, **b2**) larvae. **a1**, **b1** Ventral view of untreated and 5-HT-treated larvae. Note the enhanced sprouting and absence of compact ventral neurite bundles in the 5-HT-treated larvae. *CG* cerebral ganglion, *LNC* lateral nerve cord, *PlG* pleural ganglion

We have performed similar pharmacological manipulations during the development of the mussel *Mytilus trossulus* as described for *Platynereis* [21]. The experimental data presented in Fig. 4 demonstrate normal neurodevelopment (Fig. 4a–a2) and larval nerve elements malformed by application of 5-HT (10^−6^ M to early trochophores, up to the veliger stage) (Fig. 4b–b2). Numerous nerve processes were located chaotically in experimental larvae, reminiscent of neuronal sprouting. No compact structures, such as neurite bundles characteristic of the corresponding stage of normal development, were detected. These findings are consistent with previous hypothesis that 5-HT produced by AO neurons and released from the AO compact neuropil is involved in navigation of the long axons of early peripheral sensory cells. Further investigations to gain a deeper understanding of the action of 5-HT in axonal navigation and its consequence for nervous system formation in lophotrochozoan larvae are required.

### Involvement of neurons that secrete acetylcholine, serotonin, and FMRFamide in the development of bivalve mollusks

Immunolabeling is widely utilized to visualize differentiation in specific nerve elements of various taxa, but, unfortunately, only a limited number of commercial antibodies are applicable for invertebrates. Among these, antibodies against 5-HT and FMRF-RP are most commonly used. In addition, antibodies directed towards acetylated alpha-tubulin provide a marker for neuronal processes and some neuronal cell bodies in annelids and mollusks (personal data, [74–76]).

**Fig. 5:**
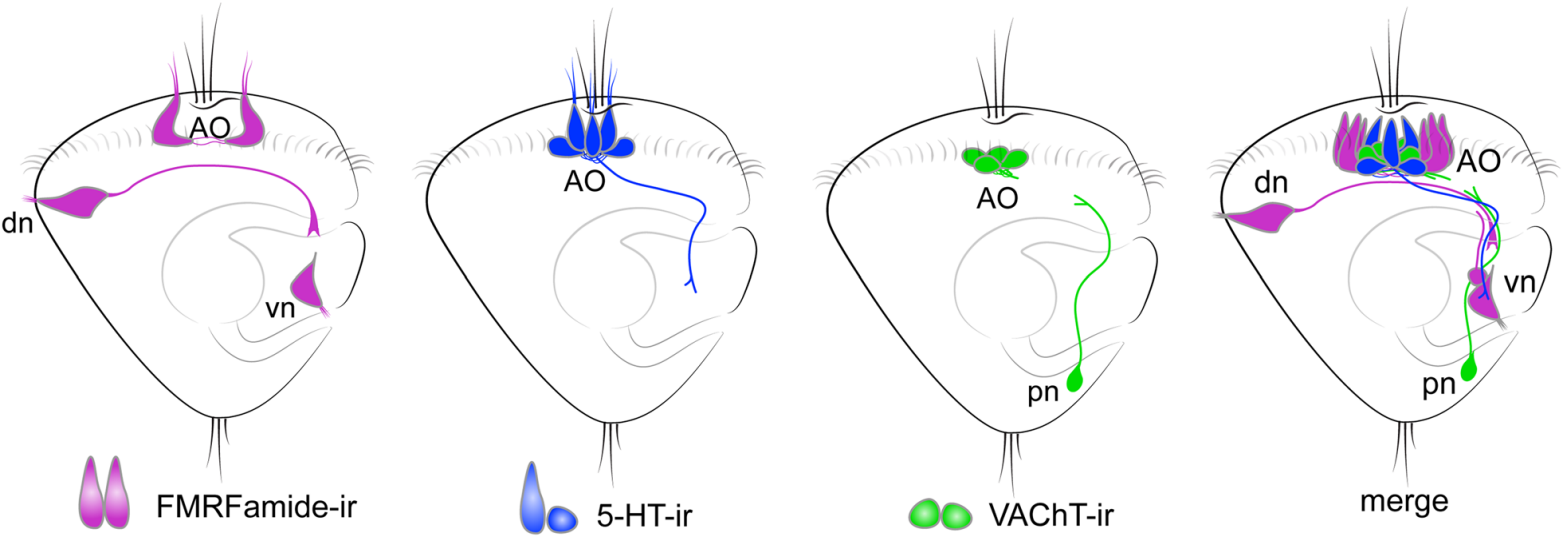
Arrangement of neurons expressing different transmitters at the veliger stage of the bivalve mollusk *Crassostrea gigas*. Only the right side of the body is shown. FMRFamide-immunoreactive fibers first grow dorso-ventrally and then turn posteriorly. 5-HT-ir fibers grow in anterior-to-posterior direction from the apical organ (AO), while VAChT-ir grows in the posterior-to-anterior direction towards the AO. Altogether, these processes form a compact neurite bundle along the ventral side of the larval body. The right and left bundles run in parallel on the respective sides of the larval body, organizing rudiments of the paired lateral nerve cords. *AO* apical organ, *dn* dorsal neurons, *vn* ventral neurons, *pn* posterior neurons (Schematic drawings adapted by Olga Kharchenko from Yurchenko et al. [14])

Histochemical reactions and immunochemical labeling against enzymes involved in biochemical degradation have been employed in attempts to visualize acetylcholine-containing (ACh-containing) regions as well as individual cells and nerve bundles; but these approaches in many cases have produced inconsistent results which were hard to interpret. In our recent study on neurogenesis in *C. gigas*, we showed that an antibody against the vesicular acetylcholine transporter (VAChT) is a reliable marker of ACh-containing elements [14]. With this antibody, we could demonstrate that at the trochophore stage, VAChT-positive reaction visualizes neurons within the AO as well as posterior neurons (Fig. 5). At the veliger stage, VAChT-ir neurons are present in cerebral and pleural ganglia and VAChT-ir fibers are found along the lateral nerve cords [14]. In addition, immunolabeling and whole-mount in situ hybridization for choline acetyltransferase were shown to identify ACh-containing elements in the central nervous system and peripheral neurons of gastropods, bivalves, and annelids [14, 26, 77–81].

## Conclusions

In summary, neurogenesis in bivalve mollusks begins with the emergence of peripheral FMRF-ir cells with their growing processes and apical 5-HT-ir sensory cells. Together, these early elements delineate the main structure of the nervous system, including the apical/cerebral ganglia and paired nerve cords. VAChT-ir cells later appear in the periphery and their processes follow the pathway already established by early “pioneer” neurons (Fig. 4). Both early 5-HT-ir and FMRF-ir fibers grow in an anterior-to-posterior direction, from the AO to the ventral portion of the larvae, while the processes of VAChT-ir cells extend in the opposite direction sending axons towards the AO and along the ventral part of the larvae. In bivalve mollusks, FMRF-ir and VAChT-ir neurites participate in the formation of nerve cords to a greater extent than 5-HT-ir neurites. At the same time, a gradient of 5-HT probably plays an important role in the navigation of FMRF-ir axons and, finally, in formation of the lateral nerve cords.

The second neuronal cord, i.e., the pedal (ventral) cord with associated pedal ganglia, appears late in bivalve development, after pediveliger settling. Further research on bivalve neurogenesis, with a particular focus on VAChT-ir structures, is required to determine the cellular compositions of the AO and the central and peripheral nervous systems of veligers in detail.

### Future perspectives

At present, many major questions and gaps remain regarding the cellular and molecular mechanisms of neurogenesis in bivalve mollusks:

1. Are some of the early neurons a prerequisite for the main ganglia of the definitive nervous system in mollusks?
2. When do ganglia fuse and what is the underlying mechanism and in response to what stimulus it starts?
3. Why does the pedal nervous system appear later in the development of bivalve mollusks and how is its development connected to that of the foot? What happens to the pedal ganglia in mollusks where the foot is resorbed (cementing species)?
4. Do mollusks have a peripheral (autonomous) nervous system?
5. How and where (placodes, neuroectoderm, and ectoderm) do neural precursors form and neuroblasts migrate?
6. What characters of the early development are common for various taxa and thus may reflect the neuroarchitecture of the last common ancestor?

## Data Availability

Not applicable.
